# Vesicular expression and release of ATP from dopaminergic neurons of the mouse retina and midbrain

**DOI:** 10.3389/fncel.2015.00389

**Published:** 2015-10-06

**Authors:** Tracy Ho, Andrew I. Jobling, Ursula Greferath, Trinette Chuang, Archana Ramesh, Erica L. Fletcher, Kirstan A. Vessey

**Affiliations:** ^1^Visual Neuroscience Laboratory, Department of Anatomy and Neuroscience, The University of MelbourneParkville, VIC, Australia; ^2^Polyclonal Antibody Development, R&D Antibody Development, EMD MilliporeTemecula, CA, USA

**Keywords:** purinergic, extracellular adenosine tri-phosphate (eATP), vesicular nucleotide transporter (VNUT), SLC17A9, dopamine, retinal neurons, substantia nigra, ventral tegmental area (VTA)

## Abstract

Vesicular nucleotide transporter (VNUT) is required for active accumulation of adenosine tri-phosphate (ATP) into vesicles for purinergic neurotransmission, however, the cell types that express VNUT in the central nervous system remain unknown. This study characterized VNUT expression within the mammalian retina and brain and assessed a possible functional role in purinergic signaling. Two native isoforms of VNUT were detected in mouse retina and brain based on RNA transcript and protein analysis. Using immunohistochemistry, VNUT was found to co-localize with tyrosine hydroxylase (TH) positive, dopaminergic (DA) neurons of the substantia nigra and ventral tegmental area, however, VNUT expression in extranigral non-DA neurons was also observed. In the retina, VNUT labeling was found to co-localize solely with TH-positive DA-cells. In the outer retina, VNUT-positive interplexiform cell processes were in close contact with horizontal cells and cone photoreceptor terminals, which are known to express P2 purinergic-receptors. In order to assess function, dissociated retinal neurons were loaded with fluorescent ATP markers (Quinacrine or Mant-ATP) and the DA marker FFN102, co-labeled with a VNUT antibody and imaged in real time. Fluorescent ATP markers and FFN102 puncta were found to co-localize in VNUT positive neurons and upon stimulation with high potassium, ATP marker fluorescence at the cell membrane was reduced. This response was blocked in the presence of cadmium. These data suggest DA neurons co-release ATP via calcium dependent exocytosis and in the retina this may modulate the visual response by activating purine receptors on closely associated neurons.

## Introduction

Extracellular adenosine 3′-triphosphate (eATP) acts as a potent signaling molecule in various tissues, mediating physiological and pathological responses on binding to cell surface purinergic receptors (Burnstock, 2007; Abbracchio et al., 2009). The mechanisms by which eATP is released continue to be heavily debated and appear to be far more varied than those described for most other neurotransmitters. ATP efflux mechanisms can be broadly classified into two types: (1) conductive mechanisms, which involve passive, osmotic diffusion into the extracellular environment via maxi-anion channels, volume-regulated ion channels, pore-forming connexins, pannexins, and P2X7-receptors and; (2) exocytotic mechanisms, which involves ATP accumulation within synaptic vesicles and active exocytosis (Lazarowski, 2012; Mutafova-Yambolieva and Durnin, 2014). In 2008, Sawada et al. identified a vesicular transporter for active accumulation of ATP within synaptic vesicles (Sawada et al., 2008). Vesicular nucleotide transporter (VNUT) protein, encoded by the solute carrier 17, member 9 (*SLC17A9*) gene, was found to be a novel member of an anion transporter family. It was identified as a mediator of active accumulation of nucleotides (i.e., ATP, ADP, and UTP) into secretory vesicles and was found to be required for exocytosis of ATP from various tissue types (Sawada et al., 2008; Iwatsuki et al., 2009; Tokunaga et al., 2010; Sathe et al., 2011; Larsson et al., 2012; Geisler et al., 2013; Oya et al., 2013; Sesma et al., 2013; Haanes et al., 2014; Harada and Hiasa, 2014).

Within the retina, ATP and its breakdown products have been implicated in modulation of a number of retinal circuits, via actions on P1 or P2 receptors (Lohr et al., 2014). Indeed, many ionotropic (P2X) and metabotropic (P2Y), ATP sensitive receptor subtypes have been characterized in the retina of the mouse (Kaneda et al., 2004; Shigematsu et al., 2007; Vessey and Fletcher, 2012; Ho et al., 2014), rat (Puthussery and Fletcher, 2004, 2006, 2007; Ward et al., 2008; Ward and Fletcher, 2009; Zhang et al., 2012), primate (Ishii et al., 2003; Puthussery et al., 2006) and human (Pannicke et al., 2000; Fries et al., 2005; Tovell and Sanderson, 2008), indicative of a role for the purinergic system in visual processing. In the brain, ATP has also been suggested to act as a neurotransmitter based on the expression of purine P2X and P2Y receptors by neurons, particularly in regions such as the hippocampus and substantia nigra (Amadio et al., 2007; Burnstock et al., 2011). Despite the possible importance of purinergic signaling within the CNS, the molecular mechanism(s) responsible for ATP release in the retina remains largely unknown.

While several lines of evidence indicate ATP release occurs via conductive mechanisms following physiological and mechanical stimuli (Newman and Zahs, 1998; Mitchell, 2001; Newman, 2003, 2015; Pearson et al., 2005; Reigada and Mitchell, 2005; Xia et al., 2012), only a few studies have focused on the possibility of a vesicular ATP release mechanism (Santos et al., 1999; Reigada and Mitchell, 2005; Loiola and Ventura, 2011). In the retina, ATP release via exocytosis has been shown using cultures of retinal glia (Loiola and Ventura, 2011; Newman, 2015), cholinergic neurons (Santos et al., 1999) and retinal pigment epithelial cells (Reigada and Mitchell, 2005). Moreover, ATP may be co-released from amacrine cells together with classical neurotransmitters, such as acetylcholine and GABA (Neal and Cunningham, 1994; Santos et al., 1999), however, the source of ATP and the mechanism of release has not been determined. Recently, *Slc17a9* expression was demonstrated in the mouse retina, suggesting that vesicular ATP release may indeed play a role in purinergic transmission (Vessey and Fletcher, 2012). However, the cellular location of VNUT and consequently the source of vesicular ATP remain to be elucidated. With the use of a novel antibody directed against the VNUT protein, the aim of the present study was to characterize the location and function of VNUT positive cells within the mouse retina and brain. The results of this study expand our understanding on the role of vesicular ATP release in purinergic neurotransmission in the central nervous system.

## Materials and methods

### Animals

All experimental procedures using animals were performed in accordance with the recommendations of the Association of Vision Research and Ophthalmology (ARVO) Statement for the Use of Animals in Ophthalmic and Vision Research and The University of Melbourne Animal Ethics Committee (Ethics #: 1112260). The protocol was approved by The University of Melbourne Animal Ethics Committee. All rats and mice were housed and maintained in plastic cages with *ad libitum* access to food and water under a 12 h:12 h light-dark cycle. Adult C57BL/6J mice (http://jaxmice.jax.org/strain/013636.html) and Dark Agouti rats (male and female; 6–8 weeks old; Animal Resources Centre, WA, Australia) were used for experimental procedures. Mice were deeply anesthetized by intraperitoneal injection of ketamine (130 mg/kg; Provet, VIC, Australia) and xylazine (27 mg/kg; Troy Laboratories, NSW, Australia) and sacrificed by cervical dislocation. Rats were deeply anesthetized by intraperitoneal injection of ketamine (130 mg/kg) and xylazine (27 mg/kg) and sacrificed using intraperitoneal injection of sodium pentobarbital (120 mg/kg; Provet).

### *Slc17a9* variant gene expression

In addition to the full length *Slc17a9* gene (NM_183161), putative protein coding isoforms have recently been predicted (transcript variants X1, XM_006500589; X2, XM_006500590; and X3, XM_006500591). In order to determine whether any of these variants were expressed in the mouse retina and brain, adult (3 month old) C57Bl6J mice were euthanized (as above) and the retina and midbrain isolated. Tissue samples were snap frozen in liquid nitrogen and stored at −80°C until use. Total RNA was isolated from retinal and midbrain tissue samples using commercial spin columns (RNeasy, Qiagen, Valencia CA) incorporating an on-column DNase I digest to remove genomic contamination. 500 ng of total RNA was reverse transcribed using a specific reverse primer (5′- GAGCAAGCAGAGCACAACTG-3′) targeting the full length *Slc17a9* gene and a commercial reverse transcription kit (Tetro, Bioline, London, UK). The subsequent template was amplified (MyTaq, Bioline) using primers (forward 5′-CTTTGGCTGGAACAAGAAGG-3′; reverse 5′-TAGTACGCCCAGAGCAAGGT-3′) that flanked the variable region in the coding sequence for the full length, X1, X2 and X3 transcript variants (NM_183161, XM_006500589, XM_006500590, XM_006500591). These primers did not distinguish between the X1 and X2 variants, as the variable region is found in the 5′ untranslated region of the gene. The amplified products were purified after agarose gel electrophoresis (Qiaquick, Qiagen) and sequenced (Australian Genome Research Facility, VIC Australia) to confirm the sequence of *Slc17a9* variants. Full length, X1, X2, and X3 transcript variants were aligned with the sequenced products to confirm identity and highlight the variable regions in the respective sequences (Clustalw, http://www.genome.jp/tools/clustalw/).

### Western blot analysis

To determine whether the detected gene transcripts coded for protein variants and also to ensure the specificity of the VNUT antibody, Western blot analysis was undertaken. Isolated mouse retinae, cortex and kidney were sonicated in homogenizing buffer (40 mM HEPES; 320 mM Sucrose; pH, 7.5) containing a cocktail of protease inhibitors (1 tablet cOmplete® per 10 mL; Roche, VIC, Australia). Homogenates were centrifuged at 7,500 g for 1 min at room temperature. The supernatants were collected and centrifuged again at 13,000 g for 15 min to separate soluble proteins from membrane bound proteins in the pellet. A mouse VNUT control peptide was produced from a plasmid (*mus musculus* solute carrier family 17, member 9, *Slc17a9* obtained as transfection ready DNA from Origene; Rockville, MD, USA) and produced *in vitro* using an EasyXpress protein Synthesis Kit (Qiagen) according to the manufacturer's specifications. The membrane-bound pellets (30 μg) and the VNUT peptide (4 μl) were re-suspended in homogenizing buffer and diluted with sample buffer (0.5 M Tris-HCl, pH 6.8; 10% SDS; 25% glycerol; 0.5% bromophenol blue; 0.5% beta-mercaptoethanol). Samples were boiled for 4 min then centrifuged at 13,000 g for 45 s. Denatured protein was loaded onto 12% Acrylamide/Bis-Tris gels along with a molecular weight marker (Odyssey®-Licor, Millenium Science, VIC, Australia). Proteins were separated on the gel by electrophoresis and transferred onto nitrocellulose membranes. The membranes were briefly stained with Ponceau S to ensure protein transfer and then rinsed with dH_2_O. Membrane-bound proteins were blocked with 1% bovine serum albumin (BSA) and 10% normal goat serum (NGS) in Tris-buffered saline with 0.05% Tween-20 (TBS-T) for 1 h on a rocker at room temperature. The membranes were then incubated with the primary antibody directed against VNUT (Guinea pig anti-VNUT; 1:3000; EMD Millipore, Temecula, CA, USA) diluted in 1% BSA and 3% NGS in TBS-T. As a peptide block control, in some instances the primary antibody directed against VNUT was pre-incubated overnight with five times the concentration of the peptide antigen prior to application to the membranes. In all instances, mouse-anti GAPDH (1:3000, Sigma-Aldrich, NSW, Australia) was applied at the same time as the VNUT antibody, as a protein loading control. After incubation in primary antibody overnight on a rocker, membranes were washed three times with TBS-T, incubated with the secondary antibodies (goat anti-guinea pig 800 at 1:1000, ImmunoReagents, Raleigh, NC, USA; goat anti-mouse 680 at 1:1000, Odyssey®-Licor) diluted in 1% BSA and 3% NGS in TBS-T for 1 h and washed again in TBS-T. The membranes were imaged using an Odyssey CLx Infrared imaging system (Odyssey®-Licor) and the same settings used for all blots including the peptide block controls.

### Immunohistochemistry

For immunohistochemistry on retinal sections, eyes were enucleated and the anterior segment and vitreous were removed. The posterior eyecups were then fixed in 4% paraformaldehyde in 0.1 M phosphate buffer (pH 7.4; PB) for 30 min and cryoprotected in graded sucrose (10%, 20%, 30%) in PB overnight. For retinal sections, the tissue was embedded in optimal cutting temperature (OCT) compound (Tissue-Tek, Sakura, CA, USA), frozen at −20°C, and sectioned transversely at 14 μm on a Microm HM550 cryostat (Thermo Scientific, Walldorf, Germany). Retinal sections were collected onto polylysine-coated slides (Thermo Scientific, VIC, Australia) and stored at −20°C. Indirect immunohistochemistry was used to determine the specific location of the guinea pig anti-VNUT antibody in sections of the transverse mouse and rat retina. For labeling of retinal sections, frozen sections were washed in PB and blocked with 10% NGS, 1% BSA and 0.5% Triton-X-100 in PB for 1 h. The sections were then incubated with primary antibodies diluted in 3% NGS, 1% BSA, and 0.5% Triton X-100 in PB overnight at room temperature. VNUT expression was assessed using a novel guinea pig primary antibody directed against VNUT/SLC17A9 (diluted 1:10000, EMD Millipore Temecula, CA, USA; Cat. No.: ABN83). Dopaminergic amacrine and interplexiform cells were labeled using a mouse primary antibody directed against tyrosine hydroxylase (TH; diluted 1:2000, Merck Millipore, Kilsyth, VIC, Australia; Cat. No.: MAB318). After washing with PB, sections were incubated with secondary antibodies (goat anti-guinea pig Alexa Fluor 488 and goat anti-mouse Alexa Fluor 594, diluted 1:500, Life Technologies Australia, VIC, Australia) for 1 h at room temperature in the dark. The sections were washed in PB, mounted with fluorescence mounting medium (Dako, NSW, Australia) and covered with a glass coverslip. Peptide block controls were obtained by pre-incubation of the VNUT primary antibody with the peptide antigen (5X excess) overnight, prior to application to the retinal sections.

For immunohistochemistry on mouse retinal wholemounts, posterior eyecups were fixed in 4% paraformaldehyde in PB for 30 min and cryoprotected. Mouse retinae in 30% sucrose in PB were exposed to repeated (3x) freeze-thaw cycles before immunohistochemical processing to improve antibody penetration. For labeling, the tissue was blocked for 1 h and incubated in primary antibodies for 4 days at 4°C. Horizontal cells were labeled using a mouse anti-calbindin D28k antibody (diluted 1:2000, Swant, Bellinzona, Switzerland; Cat. No.: 300). Cone photoreceptor terminals were labeled using the lectin Peanut Agglutinin Rhodamine conjugate (diluted 1:250, Vector Labs, Burlingame, CA, USA; Cat. No.: RL-1072). Secondary antibodies were incubated overnight at room temperature. The retinae were dissected from eyecups, mounted onto glass slides with fluorescence mounting medium (Dako) and coverslipped with the ganglion cell layer side up.

For immunohistochemistry on brain slice preparations, deeply anesthetized mice were perfused transcardially with 20 ml of 0.9% normal saline followed by the same volume of 4% paraformaldehyde in PB. Brains were removed and postfixed in the same fixative for 1 h at 4°C, and were then transferred to 10% sucrose in PB for 1 h and 30% sucrose overnight. Brains were then frozen in OCT compound, and cryostat sections were cut at 50 μm and collected in 24-well tissue culture plates (two or three sections per well) in PB. Cryostat brain sections containing the substantia nigra and VTA were incubated free-floating in guinea pig anti-VNUT (diluted 1:10000) and mouse anti-TH (diluted 1:2000) primary antibodies for 48 h at 4°C. After washing in PB, sections were incubated in secondary antibodies (goat anti-guinea pig Alexa Fluor 488 and goat anti-mouse Alexa Fluor 594, diluted 1:500) overnight at 4°C. They were then washed in PB and mounted onto glass slides in fluorescence mounting medium (Dako).

### Confocal microscopy and image analysis

Images were taken using the Zeiss LSM-5 confocal laser scanning microscope (Zeiss, Oberkochen, Germany) using 10x/0.45, 20x/0.8, 40x/1.3, and 63 x/1.4 oil-immersion objectives at a resolution of 1024 × 1024 pixels. The appropriate fluorescence filters were used (Alexa 594/Cy3: excitation 568 nm, emission filter 605/32; Alexa 488/FITC: excitation 488 nm, emission filter 522/32) for visualization of the fluorophores. Confocal settings were not altered between images of retinal sections labeled with VNUT antibody and the peptide block controls.

Confocal z-series that spanned the layers of interest in flatmounted retinas were acquired with the optimal pinhole size (1 Airy unit) as suggested by the software. In some cases, selected consecutive focal planes were merged to form a single composite image as a maximum intensity z-projection by Zeiss LSM Image Browser software (v4.2.0.121, Zeiss). Magnifications varied, with scale bars digitally added to the images by Zeiss LSM Image Browser software. Two-dimensional images were adjusted when appropriate for brightness, contrast and black levels in Adobe Photoshop CSE Version 4 (Adobe System, CA, USA).

Three-dimensional (3D) reconstruction of flatmounted retinas was performed using IMARIS software (x64, v.7.7.0 Bitplane AG, Zürich, Switzerland). Confocal z-stacks were reconstructed into 3D images in the *Surpass* mode. Background noise of each fluorescent channel was minimized individually on the *Display adjustments* panel. In some cases, images were cropped to the relevant part of the field without altering the resolution. 3D representations of retinal neurons and synaptic terminals in confocal z-stacks were created with the *Surface* function. Rendered images of different magnifications were captured using the *Snapshot* function and exported as Tiff files.

### Antibody characterization

The guinea pig anti-vesicular nucleotide transporter/SLC17A9 (EMD Millipore Temecula, CA, USA; Cat. No.: ABN83) is a novel antibody corresponding to the extracellular domain of mouse VNUT. The synthetic peptide consisting of amino acids 155–167 of murine VNUT was made at EMD Millipore (Temecula, CA, USA). New Zealand White rabbits were immunized with a linear synthetic peptide (SQKVQESERAFTY) conjugated to KLH via a C-terminal cysteine. Specificity and cellular expression of this antibody are described below in the results.

A rabbit polyclonal anti-vesicular nucleotide transporter/SLC17A9 antibody from MBL (MBL International Corporation, Des Plaines, IL, USA) was also tested. This antibody was raised to human SLC17A9 isoform 2 and was expected to cross react with rodent VNUT according to the manufacturer specifications. This antibody gave specific labeling in the mouse and rat retina that could be blocked by the associated peptide antigen and Western blot of the retina and cortex revealed binding to a single, specific band of around 70 kDa, the expected size of VNUT (Sawada et al., 2008). However, further testing indicated that this antibody did not recognize purified murine VNUT in Western blot (data not shown). In addition, sequencing of the amino acid antigen revealed that while the peptide was indeed specific for human VNUT isoform 2, it did not correlate well with the mouse VNUT sequence. Only a single amino acid out of 16 in the peptide antigen aligned within a similar region of the mouse VNUT protein and no significant similarity was found by NCBI blast comparison. Following these results we did not pursue this antibody further (data not shown).

The mouse anti-calbindin D28k recognizes a specific 28-kDa protein in Western blots of mouse brain extracts, and immunoreactivity was absent in the cerebellum of calbindin D-28K knockout mice (manufacturer's data sheet). This antibody has been shown to label horizontal cells in the retina of the mouse (Jellali et al., 2002; Vessey and Fletcher, 2012) and salamander (Zhang et al., 2006).

The mouse anti-GAPDH (clone GAPDH-71.1) recognizes a single band at ~37 kDa on Western blots corresponding to the weight of the full length GAPDH protein using A431 total cell extracts (manufacturer's data sheet). A protein band of the same size has been reported on Western blots of rat (Zabouri et al., 2011) and mouse (Vessey and Fletcher, 2012) retinal extracts.

The mouse anti-TH recognizes a protein band of approximately 59–61 kDa by Western blot of purified PC12 cell lysates (manufacturer's data sheet). This antibody has been shown to label dopaminergic neurons in the retina of the mouse (Witkovsky et al., 2008; Yoshida et al., 2011) and rat (Puthussery and Fletcher, 2006; Zhang et al., 2013), and in the midbrain of the mouse (Shaw et al., 2010) and rat (Redmond et al., 2009).

### Retinal neuron preparation for functional analysis

Dissociated neuronal cells from adult C57BL/6J mice tissues were prepared for functional analysis. For any given (*n* = 1) experiment, four retinae from two mice were isolated and placed in a HEPES buffered physiological saline solution (PSS; NaCl 137 mM, KCl 2.5 mM, HEPES 10 mM, D-glucose 28 mM, NaH_2_PO_4_ 1.25 mM, MgCl_2_ 1 mM, and CaCl_2_ 2 mM). Retinal neurons were gently dissociated using a neural dissociation kit (MACs Miltenyi Biotec), according to the manufacturer's specifications and then resuspended in PSS.

### ATP release assay

To assess whether a depolarizing stimuli induced ATP release from dissociated retinal neurons (*n* = 5 separate neuronal preparations), cells were re-suspended at a 1 × 10^6^ cells/mL in PSS and maintained on ice. Using the ENLITEN® ATP Assay (Promega, Alexandria, NSW, Australia; Cat# FF2000), ATP release from dissociated neurons was determined according to manufacturer's protocol. Briefly, 5 μL of ATP (10^−14^–10^−5^M) was combined with 50 μL of rL/L reagent and luminescence measured over a 20 s epoch to generate a standard curve. To assess ATP release from the retina, neuronal samples were heated to 37°C and 10 μL of neuronal preparation in PSS and 50 μL of rL/L was combined with either: 1) 10 μL of PSS to assess basal ATP release or 2) 10 μL of a neuron depolarizing, 55 mM K^+^-PSS high potassium, low sodium PSS (final NaCl 27 mM, KCl 55 mM, HEPEs 10 mM, D-glucose 28 mM, NaH_2_PO_4_ 1.25 mM, MgCl_2_ 1 mM, and CaCl_2_ 2 mM) to assess neuronal ATP release; or 3) 10 μL of 55 mM K^+^-PSS to depolarize neuronal preparations where voltage-gated calcium channel-dependent synaptic transmission was blocked by pre-incubated with 400 μM Cadmium (Cd^++^).

### Fluorescent dye loading and confocal imaging *in vitro*

Dissociated retinal neurons were re-suspended in PSS containing 0.5% BSA at 5 × 10^6^ cells/mL and incubated with the guinea pig anti-VNUT antibody (1:100) for 15 min at 4°C and washed with 20 mL of PSS. Cells were centrifuged at 130 g for 10 min at 4°C and re-suspended in 4 mL of PSS containing 0.5% BSA. A secondary goat anti-guinea pig Alexa 594 (1:500, Life Technologies, Cat#A-11076) was applied for 15 min at 4°C in conjunction with fluorescent dyes for ATP-containing vesicles (Mant-ATP 50 μM; Life Technologies, Cat#M-12417 or Quinacrine 10 μM, Sigma-Aldrich Australia, Cat#Q3251) and dopamine containing vesicles (Fluorescent false neurotransmitter, FFN102 50 μM; Abcam, Sapphire Biosciences, NSW, Australia, Cat#AB120866). The fluorescent dyes for the ATP-containing and dopamine-containing vesicles excite in the 488 and 380 nm ranges, respectively (Dou et al., 2012; Rodriguez et al., 2013). Cells were washed in 20 mL of PSS, centrifuged at 130 g for 10 min at 4°C and re-suspended at 5 × 10^7^ cells/mL and placed on ice until imaging. A cell volume of 200 μL was applied to a glass coverslip-bottomed petri dish and the sample covered with a small glass cover slip. Images were collected using a X63 oil objective, with X7 times zoom on an inverted SP5 confocal laser scanning microscope (Leica microsystems, North Ryde, NSW, Australia), with the chamber temperature set at 37°C. Scan speed was optimized for image quality and still shots collected at 100 Hz and movies collected at 1400 Hz.

To assess ATP release from labeled cells, neuronal preparations in PSS were imaged over a 1 min epoch in response to: (1) No treatment, to assess for fluorescence bleaching and basal vesicle release; (2) Administration of PSS to assess for fluorescence bleaching and basal vesicle release in response to changes in volume; (3) A neuron depolarizing, 55 mM K^+^-PSS to assess vesicle release; or (4) 55 mM K^+^-PSS to depolarize neuronal preparations where voltage-gated calcium channel-dependent synaptic transmission was blocked by pre-incubation with 400 μM Cadmium (Cd^++^). In general, treatments were administered within the first 15–20 s of imaging and for the purposes of analysis, slight differences in the start time were normalized by considering only the 10 s epoch before treatment as the start time. At least *n* = 5–10 cells were analyzed per treatment from *n* = 6 neuronal preparations. Changes in fluorescence intensity of puncta near the cell membrane were assessed over time using a customized Image J 1.43 Freeware script (NIH, USA) and normalized to average intensity at baseline over the first 10 s prior to treatment.

### Statistics

Results are expressed as the Mean ± Standard Error of the Mean (SEM). Statistical analysis was performed using Graphpad Prism v6 (San Diego, CA, USA) and One-Way ANOVA used to assess the effect of treatment conditions. Statistical significance between treatments is indicated by ^*^ for *p* < 0.05.

## Results

### Multiple isoforms of the *Slc17a9* gene are expressed in the mouse retina and brain

**Figure 1:**
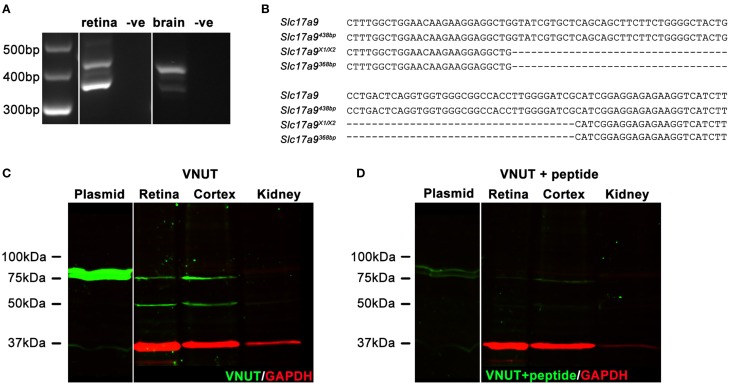
**Two native isoforms of Slc17a9/VNUT are expressed in the mouse retina and brain**. *Slc17a9* isoform transcript expression was assessed in mouse retina and brain. Two protein coding *Slc17a9* isoform transcripts were identified. **(A)** A 438 bp PCR product band (upper band), representing full length *Slc19a9*, and a 368 bp band (lower band), representing isoforms X1/X2 was apparent in both retina and midbrain. **(B)** PCR product specificity was confirmed by sequence alignment of a 120 bp region spanning the variable region. **(C)** Western blot analysis of purified full length VNUT protein incubated with guinea pig polyclonal anti-VNUT antibody showed an immunoreactive band at ~75 kDa (plasmid, green). In contrast, two isoforms of native VNUT were identified in retinal and cortical homogenates at ~50 kDa and ~75 kDa (green). **(D)** VNUT immunoreactivity was blocked by pre-incubation with excess peptide antigen. Little/no VNUT labeling was detected in mouse kidney homogenates. All immunoblots were co-labeled with GAPDH as a protein loading control (red).

In addition to full length *Slc17a9* sequence, an additional three potential protein coding isoforms have been predicted (X1, X2, and X3). Expression of *Slc17a9* and the putative isoforms were assessed in mouse retina and midbrain (Figures 1A,B). Using primers that spanned the variable region, a 438 bp PCR product (Figure 1A, upper band) and a smaller 368 bp PCR product (Figure 1A, lower band) were detected in both retina and midbrain samples. When sequenced, the 438 bp product showed 100% identity with the full length *Slc17a9* sequence (Figure 1B, upper two gene sequences), while the smaller product showed complete identity to the X1 and/or X2 isoforms (Figure 1B, lower two gene sequences). As the X1 and X2 variants differ in the 5′ untranslated region of the gene, the expression of these isoforms could not be distinguished. Respective negative controls for retina and midbrain samples discounted the possibility of genomic contamination (Figure 1A).

To determine whether the detected gene transcripts coded for protein variants, Western blot analyses on purified mouse VNUT/SLC17A9 protein and homogenates of mouse cortex, retina and kidney were performed. The novel guinea pig antiserum recognized the full length purified VNUT protein, labeling a band of ~75 kDa (Figure 1C, left blot, plasmid). In contrast, the reactivity was negligible when the antibody was pre-incubated with excess antigen peptide (Figure 1D, left blot, plasmid), indicating that the antibody was specific for full length mouse VNUT protein. In addition, the absence of GAPDH signals on these immunoblots suggests that the *in vitro* production of VNUT peptide from plasmid resulted in little contaminating peptide expression, reducing the chances of false labeling. In the retinal and cortical homogenates, two bands of ~75 kDa and ~50 kDa were recognized by the VNUT antibody (Figure 1C, right blot). The larger band corresponds to the predicted molecular weight reported in previous studies for full length VNUT (Sawada et al., 2008; Larsson et al., 2012; Hiasa et al., 2014). The smaller ~50 kDa band likely represents the smaller isoform X1/X2. Both the ~75 kDa and ~50 kDa bands were eliminated when the antibody was pre-incubated in excess peptide antigen (Figure 1D, right blot), suggesting specific reactivity with native VNUT from mouse retina and cortex. No labeling was observed in the kidney membrane preparations (Figure 1C), indicating that VNUT expression is likely very low or may be absent in mouse kidney.

### VNUT is expressed by dopaminergic neurons in the mouse retina and midbrain

**Figure 2:**
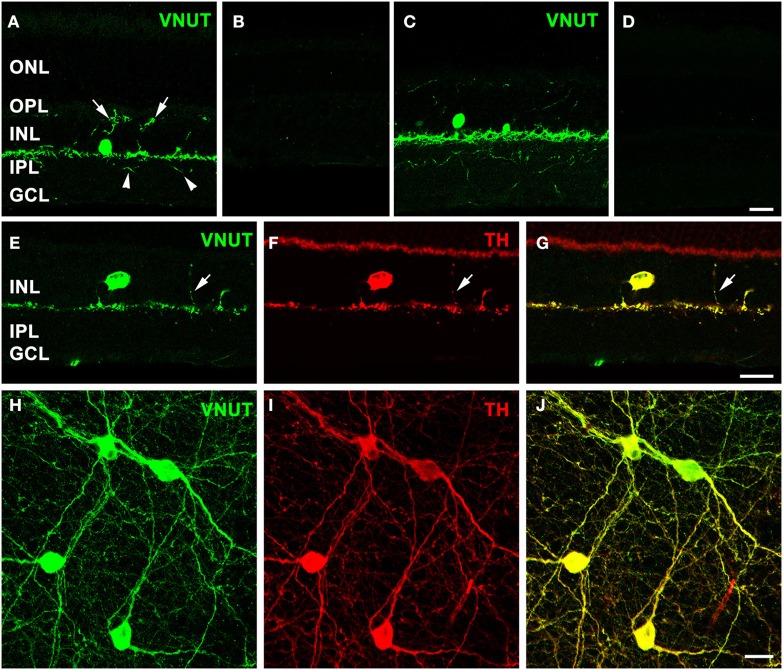
**VNUT-immunoreactive cells co-localize with tyrosine-hydroxylase positive dopaminergic amacrine/interplexiform cells**. Vertical cryostat sections of **(A)** adult mouse and **(C)** rat retinae expressed VNUT-IR in an amacrine/interplexiform cell (IPC) type in the INL. Pre-incubation of the VNUT antibody with excess peptide antigen resulted in no immunoreactivity, confirming the specificity of the antibody in the **(B)** mouse and **(D)** rat retinas. **(E–J)** VNUT immunoreactivity (green) was exclusively expressed in tyrosine hydroxylase-labeled dopaminergic amacrine/IPC cells and processes (red) as shown in a vertical section **(E–G)** and flatmounted mouse retina **(H–J)**. Arrows **(A, E–G)** indicate interplexiform processes co-labeled with VNUT and TH extending toward the OPL. Arrowheads **(A)** indicate processes extending towards the IPL. ONL, outer nuclear layer; OPL, outer plexiform layer; INL, inner nuclear layer; IPL, inner plexiform layer; GCL, ganglion cell layer. Scale bars = 20 μm.

Immunolabeling performed on a vertical section of the adult mouse retina with the antibody against VNUT revealed selective expression in somata in the proximal portion of the inner nuclear layer (INL) and in labeled processes arising from the base of somata adjacent to the INL/inner plexiform layer (IPL) border (Figure 2A). In addition, fine VNUT immunoreactive (VNUT-IR) processes were found to extend deeper into the IPL (arrowheads), as well as traverse the INL and ramify in the outer plexiform layer (OPL; arrows). These processes usually branched from the primary dendrites in sublamina 1 of the IPL (Figure 2A), but occasionally they arose from the cell somata. Labeling was completely abolished with the pre-incubation of the peptide antigen control (Figure 2B). Similar labeling patterns were observed in the adult rat retina with the VNUT antibody (Figure 2C) and with the pre-adsorption of the antigen peptide (Figure 2D).

The morphology and location of the retinal VNUT-IR cells closely resembled that of dopaminergic amacrine/interplexiform cells (IPCs; Nguyen-Legros et al., 1997; Witkovsky, 2004). In order to confirm this, VNUT expressing cells were co-labeled with TH, the rate-limiting enzyme for dopamine production that is specifically expressed by DA cells (Brecha et al., 1984; Nguyen-Legros et al., 1984; Oyster et al., 1985). Double-immunolabeling on a vertical section of mouse retina showed that VNUT-IR was specifically expressed in the sparse TH-positive cells in the INL and their processes that stratify along the outer margin of the IPL (Figures 2E–G). In addition, a VNUT-IR interplexiform process extending distally toward the OPL was co-localized with TH (Figures 2E–G, arrows). Flatmount preparations imaged at the level of the INL and IPL showed exclusive overlap between VNUT (Figure 2H) and TH (Figure 2I) labeling of DA cell somata and their dense network of processes in sublamina 1 of the IPL (Figure 2J). This indicates that VNUT is expressed in all dopaminergic amacrine/IPCs in the mouse retina.

**Figure 3:**
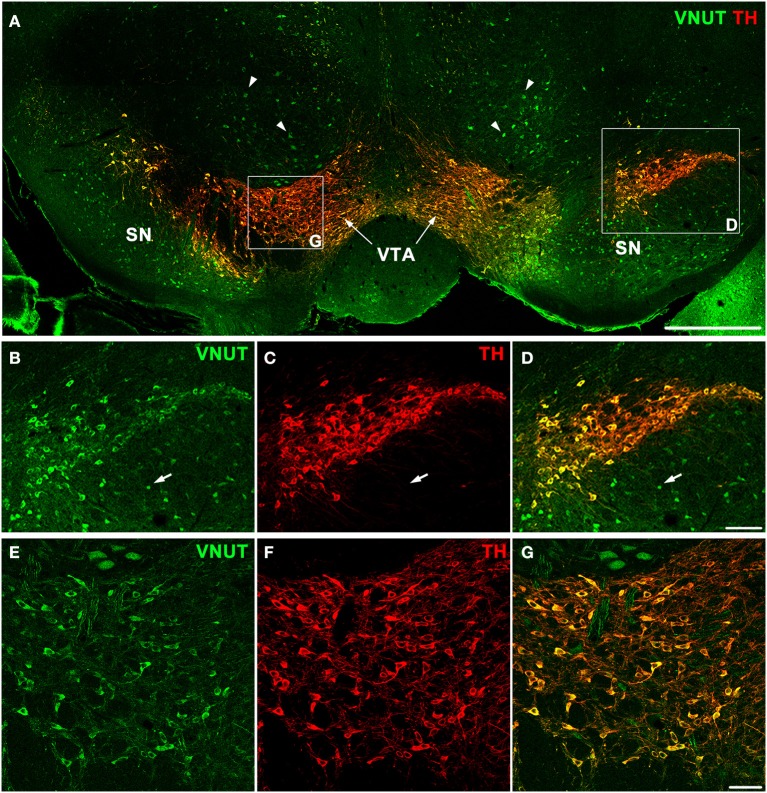
**VNUT-immunoreactive cells co-localize with tyrosine-hydroxylase positive dopaminergic neurons in the mouse substantia nigra and ventral tegmental area. (A)** A coronal section of the mouse midbrain co-labeled with VNUT (green) and Tyrosine Hydroxylase (TH, red) showing co-localization on dopaminergic neurons in the substantia nigra (SN) and ventral tegmental area (VTA). Arrowheads indicate VNUT^+^TH^−^ cells. **(B–D)** A magnified view of the SN (right inset) showing all TH-positive cells (red) are also positive for VNUT (green). Arrows in **(B–D)** indicate VNUT^+^TH^−^ cells. **(E–G)** A magnified view of the VTA (left inset) showing VNUT expression (green) in all TH-positive cells (red). VNUT expression was not restricted to DA neurons and was found in many TH-negative cells throughout this region of the brain. Scale bars = 500 μm **(A)**, 100 μm **(B–D)** and 50 μm **(E–G)**.

In addition to the retina, dopaminergic neurons are located in the substantia nigra and ventral tegmental area (VTA) of the midbrain, which is an area involved in motor, cognitive and emotion control (Cachope and Cheer, 2014). To determine whether VNUT was expressed in different populations of DA neurons of the CNS, VNUT and TH co-labeling was performed on coronal mouse brain sections (Figure 3A). All TH-positive DA cells in the substantia nigra (Figures 3B–D) and VTA (Figures 3E–G) were VNUT-positive. However, VNUT expression was not restricted to DA neurons, such that VNUT^+^TH^−^ cells were found in the substantia nigra (Figures 3B–D, arrows) and other regions of the brain (Figure 3A, arrowheads), suggesting a heterogeneity of vesicular ATP-accumulating cells in the brain. Together, these findings signify that the machinery required for vesicular ATP release is present on DA neurons in different regions of the CNS.

### In the retina VNUT-IR IPC processes are closely associated with horizontal cells and cone photoreceptors

**Figure 4:**
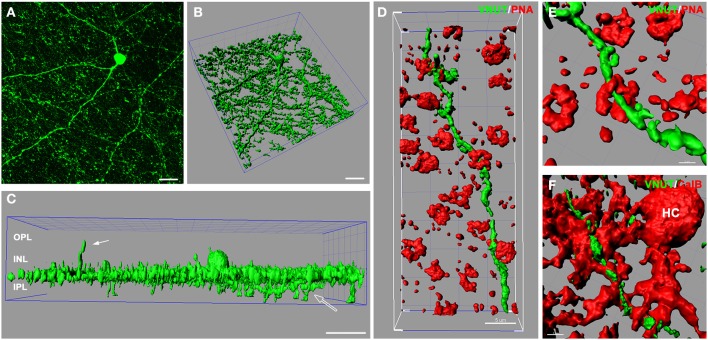
**VNUT-immunoreactive amacrine/interplexiform cell processes are closely associated with cone photoreceptor terminals and horizontal cells in the outer retina. (A)** Confocal z-stack of a retinal flatmount immuno-labeled with VNUT, showing specific expression in a cell in the inner retina with long primary dendrites and fine processes. **(B)** Three-dimensional (3D) reconstruction of the confocal z-stack in **(A)** showing a dense network of processes extending laterally beneath the cell soma. **(C)** Side projection of the 3D reconstructed VNUT-positive cell showing that most processes are distributed in a narrow horizontal plane at the distal portion of the IPL. The white solid arrow indicates an interplexiform process reaching toward the outer retina. The open arrow indicates processes extending into the proximal portion of the IPL. **(D)** Three-dimensional (3D) reconstruction of a confocal z-stack showing a VNUT interplexiform process (green) in the OPL closely associated with cone photoreceptor terminals labeled with peanut agglutinin (PNA, red). **(E)** A magnified view of a VNUT interplexiform process (green) in close association with a cone terminal (red). **(F)** 3D reconstruction of a flatmount confocal z-stack co-labeled with VNUT (green) and the horizontal cell (HC) marker, calbindin (red). The VNUT interplexiform process was found in close apposition with horizontal cell processes in the OPL. OPL, outer plexiform layer; INL, inner nuclear layer; IPL, inner plexiform layer. Scale bars = 20 μm **(A)**, 30 μm **(B,C)**, 5 μm **(D)**, 2 μm **(E)**, and 3 μm **(F)**.

In the retina, amacrine/interplexiform cells (IPCs) have been extensively reported to form close contacts (synaptic and close appositions) with photoreceptors, horizontal and bipolar cells within the OPL, providing a centrifugal pathway from the inner to the outer plexiform layers via the release of dopamine (Dowling and Ehinger, 1975; Kolb et al., 1990), GABA (Kolb et al., 1991; Witkovsky et al., 2008; Dedek et al., 2009) and glycine (Pow and Hendrickson, 1999; Haverkamp and Wassle, 2000). To better illustrate VNUT-IR processes at multiple levels of the neural retina, three-dimensional reconstruction of retinal flatmounts immunolabeled with VNUT were performed. The three-dimensional top view of the confocal acquired flatmount image (Figure 4A) shows many VNUT varicosities at the border of the INL and IPL, directly beneath the VNUT cell soma (Figure 4B). In addition to the dense plexus that was largely restricted to the outermost layer of the IPL (sublamina 1), VNUT processes were found to extend to the inner sublaminae of the IPL (open arrow) and distally across the INL toward the OPL (Figure 4C, solid arrow). This data further verifies that VNUT is expressed in IPCs in the mouse retina.

To determine the cellular associations of VNUT-IR amacrine/IPCs in the OPL, three-dimensional reconstructions of confocal acquired flatmounts co-labeled with VNUT and the cone photoreceptor label, peanut-agglutinin (PNA), or the horizontal cell label, calbindin were examined. VNUT-IR amacrine/IPC distal processes were found in close association with PNA-labeled cone photoreceptor terminals (Figure 4D), such that VNUT-IR profiles were found immediately beneath and extending through cone terminals (Figure 4E). VNUT-IPC distal processes were also found in close apposition to calbindin-labeled horizontal cells, showing intricate contacts with the plexus of horizontal cell processes in the OPL (Figure 4F). These findings suggest that VNUT-expressing amacrine/IPCs have the capacity to interact with photoreceptors and horizontal cells in the outer retina.

### ATP is released from dopaminergic neurons of the retina

**Figure 5:**
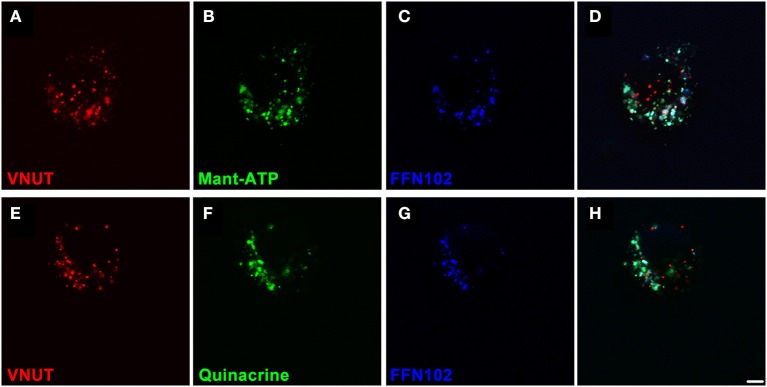
**VNUT-immunoreactive dissociated retinal neurons co-load fluorescent ATP- and dopamine-markers in vesicles**. Freshly dissociated retinal preparations were labeled for VNUT-immunoreactivity (red) and incubated with fluorescent ATP markers (green) and the fluorescent dopamine marker (FFN102, blue). **(A–D)** VNUT-positive neurons (**A**, red) co-expressed the fluorescent ATP marker Mant-ATP (**B**, green) and the dopamine fluorophore FFN102 (**C**, blue) in discrete puncta (**D**, co-localized). **(E–H)** Similarly VNUT-positive neurons (**E**, red) co-expressed the fluorescent ATP marker quinacrine (**F**, green) and the dopamine fluorophore FFN102 (**G**, blue) in discrete puncta (**H**, co-localized). Within these cells, the fluorescent ATP marker puncta co-localized with the dopamine fluorophore, FFN102, suggesting co-loading of ATP and dopamine in vesicles within VNUT-positive neurons. Scale bar = 2.5 μm.

In order to confirm that VNUT was involved in the accumulation and release of ATP within dopaminergic amacrine/IPCs, a series of functional assays were undertaken using freshly dissociated retinal preparations. Neuronal preparations were labeled for VNUT-IR, fluorescent ATP markers (Mant-ATP or quinacrine) and the fluorescent dopamine marker (FFN102) and viewed on a confocal microscope (Figure 5). VNUT-positive neurons were infrequent in the retinal preparation, confirming the limited expression in retinal vertical sections (Figure 2A). Those cells expressing VNUT were found to load the fluorescent ATP markers, Mant-ATP (Figures 5A–D) and quinacrine (Figures 5E–H), in discrete puncta. Quantitative analysis indicated there were slightly more VNUT positive puncta than fluorescent ATP puncta within each cell (2778 ± 167 vs. 2399 ± 36 puncta/100 μm^2^ of cell; *n* = 12 cells; Student's *t*-test, *p* = 0.03) and that 74 ± 1% of the VNUT puncta were co-localized with ATP fluorescence, suggesting that some VNUT positive/ATP negative puncta exist, perhaps representing early vesicles. Within these cells also, 80 ± 8% of the dopamine fluorophore, FFN102 puncta co-localized with the fluorescent ATP marker puncta (*n* = 12 cells), suggesting co-loading of ATP and dopamine in vesicles within VNUT-positive neurons. However, detailed analysis at the ultrastructural level would be required to confirm this. All cells that were positive for VNUT were also positive for fluorescent ATP- and the dopamine-marker.

**Figure 6:**
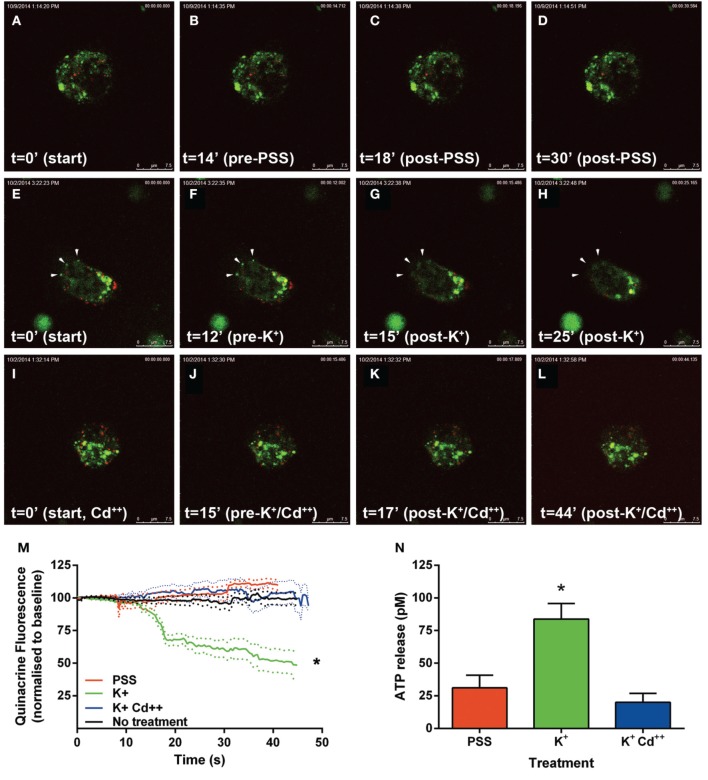
**ATP is released by calcium dependent exocytosis from VNUT positive retinal neurons**. Changes in ATP-marker fluorescence intensity near the cell membrane were assessed as an indicator of ATP release under various treatment conditions. Quinacrine (green) was used as this ATP-marker was the most stable. Retinal neurons were immuno-labeled with VNUT (red). **(A–D)** Images collected and shown over a 30 s interval under basal conditions, when vehicle (PSS) was added at 15 s. Fluorescence intensity near the membrane was not altered over the recording interval (**M**, red line, *n* = 8 cells). **(E–H)** Images collected over a 25 s interval under depolarizing-conditions, where 55 mM K^+^-PSS added at 13 s. Quinacrine fluorescence at the cell membrane reduced significantly within a second after stimulation (**M**, green line, *n* = 10 cells). Arrowheads indicate a reduction of ATP-marker fluorescence at the cell membrane. **(I–L)** Images collected over a 44 s time interval under depolarizing-conditions in the presence of cadmium Cd^++^ (55 mM K^+^-PSS added at 16 s). There was no significant change in fluorescence intensity near the membrane in the presence of Cd^++^ (**M**, blue line, *n* = 10 cells). **(N)** ATP release from the dissociated retinal cells (*n* = 5 separate neuronal preparations) was determined under basal (PSS, red), depolarizing stimuli (K^+^, green) and depolarizing stimuli following pre-treatment with cadmium (K^+^Cd^++^, blue). The level of extracellular ATP was significantly increased with the addition of K^+^-PSS, while blockade of voltage-gated Ca^++^ channels with Cd^++^ showed comparable levels of ATP release to the basal condition. Scale bars = 7.5 μm. PPS, physiological saline solution; K^+^, potassium (55 mM); Cd^++^, Cadmium (400 μM). ^*^p < 0.01.

Following the above phenotypic characterization, the capacity of these dopamine/VNUT-containing cells to release ATP was assessed by quantifying changes in ATP-marker fluorescence intensity near the cell membrane (Figure 6). For these experiments, quinacrine was used as this ATP-marker was the most stable when no treatment was applied, specifically fluorescence intensity did not reduce significantly over the recording interval when quantified (Figure 6M, black line, *n* = 8 cells). Similarly, under basal conditions, when a volume of PSS was added to account for slight defocus changes due to volume alterations, fluorescence intensity did not reduce significantly over the recording interval (Movie 1-PSS; Figures 6A–D,M, red line, *n* = 8 cells). In order to stimulate ATP release, high K^+^/low Na^++^ conditions (55 mM K^+^-PSS) were used and found to reduce ATP-marker fluorescence at the cell membrane within a second after addition (Movie 2-highK-1 and Movie 3-highK-2; arrows in Figures 6E–H). The change in fluorescence was significantly reduced to 50% of baseline (Figure 6M, green line, *n* = 10 cells). This K^+^-PSS induced-change in ATP-marker fluorescence was blocked in the presence of cadmium, suggesting that this response was voltage gated Ca^++^ channel dependent exocytosis (Movie 4-highK-Cd; Figures 6I–J,M, *n* = 10 cells). Finally, ATP release was quantified in this dissociated retinal cell population using a commercial ATP assay (ENLITEN® ATP Assay; Figure 6N). After addition of vehicle (PSS), the basal release of ATP was determined to be 25 pM over a 20 s epoch. Application of the depolarizing solution (55 mM K^+^-PSS) induced a significant increase in ATP release to 75 pM (*n* = 5 separate neuronal preparations, One-Way ANOVA, *p* < 0.05). This high potassium-induced increase in ATP release was blocked when the retinal cell preparation was pre-treated with cadmium. This suggests that in response to a depolarizing stimulus, ATP release from retinal preparations relies on Ca^++^ dependant exocytosis. Together these findings suggest that dopaminergic neurons of the retina express VNUT, load ATP into vesicles and when stimulated, release ATP via exocytosis.

## Discussion

In this study, VNUT expression was characterized in the mouse retina and brain based on RNA transcript, protein and functional analysis. Our results demonstrate, for the first time, that VNUT expression is apparent in DA-cells of the rodent retina and midbrain suggesting that ATP is stored and released by way of exocytosis from DA cells across the CNS. In the retina, VNUT-IR amacrine/IPC processes were closely associated with cone photoreceptor terminals and horizontal cell processes, suggesting that these cells may modulate outer retinal processing via regulated exocytosis of ATP.

### Two native isoforms of VNUT occur in the mouse retina and brain

In addition to a full length *Slc17a9* transcript (NM_183161), putative protein coding isoforms based on automated computational analysis have recently been predicted (X1, XM_006500589; X2, XM_006500590; X3, XM_006500591). Our data indicate that two protein coding mRNA variants are expressed in the mouse central nervous system, with products corresponding to the full length *Slc17a9* and X1/X2 isoform identified. The putative *Slc17a9* X3 isoform was not detected in the retina or midbrain. In line with the presence of these gene transcripts, two protein bands of ~75 and ~50 kDa were isolated in mouse retinal and cortical preparations using Western blot. Based on the sequence location of the antigen peptide, the novel guinea pig VNUT antibody used should recognize both full length and X1/X2 protein, but not X3 protein. The 75 kDa band correlates with the molecular weight reported previously for full length VNUT (Sawada et al., 2008; Larsson et al., 2012; Hiasa et al., 2014), whereas the smaller ~50 kDa band likely represents isoform X1/X2. Further work is necessary to identify whether these isoforms play differential roles in modulating synaptic vesicle loading and release. In addition, if there is cell specific isoform expression across differing cell types within the central nervous system, this may unveil further complexity in the purinergic neurotransmission system.

### VNUT in the midbrain

Co-localization of dopamine with other transmitters is a common phenomenon in the CNS; GABA and glutamate are expressed in subpopulations of neurons of the substantia nigra and VTA, respectively (Kaneko et al., 1990; Campbell et al., 1991; Sulzer et al., 1998). Furthermore, ATP and dopamine co-transmission from neurons in the midbrain has been reported (Krügel et al., 2001, 2003). Our results indicate that all DA neurons in the substantia nigra and VTA of the mouse midbrain co-express VNUT, suggesting that DA neurons are indeed a source of extracellular ATP and thus its bioactive degradation products such as ADP and adenosine. These purinergic ligands would mediate their actions on purine-receptors (P1 and P2) which have been found to be expressed pre-synaptically on DA neurons and also co-expressed with dopamine receptors on neurons in brain regions receiving DAergic input (Amadio et al., 2007; Morin and Di Paolo, 2014). As DA neurons in the midbrain modulate a broad range of behaviors, such as motor control, motivation and response to rewards (Wise, 2008; Joshua et al., 2009; Schultz, 2013; Cachope and Cheer, 2014), it is likely that purinergic neurotransmission plays a role in these pathways. Similarly, purinergic transmission is likely to have a role in neurological disorders that involve dysregulation of DAergic neurons in the substantia nigra, such as Parkinson's disease (Benazzouz et al., 2014). This proposal is consistent with the evidence for the therapeutic effect of adenosine receptor antagonists in the treatment of this disease (Kulisevsky and Poyurovsky, 2012; Jenner, 2014). VNUT-IR was not restricted to DA cells, but was also expressed by non-DA neurons within the substantia nigra. As VNUT has been shown to co-localize with glutamatergic neurons in the hippocampus (Larsson et al., 2012), further research is required to elucidate the cellular expression profile of non-DAergic/VNUT positive cells in the midbrain. The discovery of VNUT-IR within the substantia nigra and VTA provides a new marker for assessing functional subpopulations of cells in these brain areas.

### VNUT in the retina

Analogous to the brain, VNUT expression was found in all TH-positive somata and processes in the retina, indicating that ATP release from DA neurons may be a common feature across the CNS. The DA amacrine/IPCs were the only cell class in the retina to express VNUT, suggesting that in addition to dopamine and GABA (Hirasawa et al., 2012), these cells also co-release ATP. Further functional analysis of these VNUT-IR cells indicated that dopamine and ATP are likely co-expressed in vesicles within these cells and released via calcium dependent exocytosis upon treatment with a depolarizing stimulus. Previous work suggests that there are at least two classes of DA neuron in the mammalian retina (Nguyen-Legros et al., 1997; Zhang et al., 2007). Morphologically, these cells have somata in the INL, directly adjacent to the IPL and give rise to three dendritic plexuses, two in the IPL in strata 1 and 3, and one in the OPL, an interplexiform-process (Hirasawa et al., 2012). Within the OPL, these cells modulate photoreceptor terminals, horizontal and bipolar cells, providing centrifugal input from the inner to the outer retina (Dowling and Ehinger, 1975; Frederick et al., 1982).

Based on DA cell morphology and DA receptor distribution it is anticipated that DA modulates cell activity in the outer retina by both synaptic and extrasynaptic neurotransmission (Dacey, 1990; Kolb et al., 1990; Savy et al., 1995). Evidence for extrasynaptic transmission comes primarily from the finding that DA-IPC processes do not necessarily correlate with the expression pattern of DA receptors (Bjelke et al., 1996; Witkovsky, 2004). Similar mechanisms of action may apply for ATP release in the OPL. Evidence for synaptic neurotransmission comes from the close proximity of VNUT-IR IPC varicosities with horizontal cell processes and cone photoreceptor terminals, which express ATP sensitive, P2X-receptors (P2X3-R, P2X4-R, P2X7-R; Puthussery and Fletcher, 2004; Puthussery et al., 2006; Vessey and Fletcher, 2012; Ho et al., 2014). As ATP would be rapidly hydrolyzed to ADP, protons and adenosine by enzymes located between synapses in the OPL (Puthussery et al., 2006; Puthussery and Fletcher, 2007), it would be imperative that traditional synaptic transmission occurs for these ATP sensitive receptors to be activated. Extrasynaptic purinergic transmission, mediated by adenosine on P1 receptors is also a likely mode of action. Indeed recent evidence indicates that A2a adenosine receptors are expressed by photoreceptors and activation of these receptors increases photoreceptor coupling, opposing the action of dopamine on DR4-receptors in modulating the adaptation state of the retina (Li et al., 2013). As there is also evidence that dopamine reduces the receptive field size of horizontal cells by uncoupling gap junctions in this cell class in various species (Hampson et al., 1994; He et al., 2000; Reitsamer et al., 2006; Zhang et al., 2011), it is tempting to speculate that ATP may share similar roles with dopamine in setting the gain of the retina. Interestingly, a second, moderate speed (200 ms) extrasynaptic role for ATP appears to exist, whereby the rapid degradation of ATP to ADP and protons, increases the pH of the synaptic cleft providing feedback to horizontal cells and cones (Vroman et al., 2014). Thus, vesicular release of ATP in the OPL may mediate fast synaptic transmission via P2X-and P2Y-receptors, moderate speed modulation via pH-regulation and slow diffuse activation of P1-adenosine sensitive receptors, thereby playing a myriad of roles in modulating photoreceptors, horizontal cells and bipolar cells.

Given the widespread distribution of multiple purinergic receptor subtypes in the OPL and throughout all layers of the IPL in the retina (Puthussery and Fletcher, 2004; Puthussery et al., 2006; Ward et al., 2008; Vessey and Fletcher, 2012; Zhang et al., 2012; Ho et al., 2014), it was surprising to find that vesicular ATP storage and release was restricted to a single cell class that accounts for less than 1% of the retinal cell population (Gustincich et al., 1997; Jeon et al., 1998). One possibility is that other retinal cells with the potential for ATP release, such as cholinergic amacrine cells (Neal and Cunningham, 1994; Santos et al., 1999), horizontal cells (Vroman et al., 2014) and Müller glial cells (Newman and Zahs, 1998; Newman, 2003, 2015), may express as yet unidentified vesicular ATP transporters. Given the close phylogenetic relationships and structural similarities between SLC17A9 and the other members of the SLC17 gene family (Sreedharan et al., 2010; Reimer, 2013), and the fact that not all of these transporters have been functionally characterized, it is highly likely that other vesicular ATP transporters may also exist. In addition to the existence of other ATP transporters, contributions from conductive mechanisms such as pannexin, connexin and Cl^−^ anion channels cannot be ruled out (Lazarowski, 2012; Mutafova-Yambolieva and Durnin, 2014; Vroman et al., 2014). Thus, while our data suggest that DA-amacrine/IPCs are a source of vesicular ATP released by exocytosis in the mouse retina, it is possible that other mechanisms of release may also occur.

## Conclusion

In summary, this study presents evidence for a source of vesicular released ATP in the mouse retina and midbrain. We demonstrated that VNUT is expressed by DA-amacrine/IPCs in the retina, and DA as well as non-DA neurons in the midbrain. In addition to dopamine and GABA, our results suggest that retinal-IPCs may modulate outer retinal processing via the release of ATP at contact sites with horizontal cell processes and photoreceptor terminals. These results provide further evidence for a role of purines in neurotransmission in the retina and the brain.

## Author contributions

All authors had full access to all the data in the study and take responsibility for the integrity of the data and the accuracy of the data analysis. Study concept and design: KV, EF. Acquisition of data: TH, KV, AJ, UG. Analysis and interpretation of data: TH, KV, AJ, UG, EF. Drafting of the manuscript: TH, KV, EF. Critical revision of the manuscript for important intellectual content: TH, KV, AJ, UG, EF, TC, AR. Statistical analysis: KV. Obtained funding: EF, KV. Administrative, technical and material support: EF. Study supervision: EF, KV.

### Conflict of interest statement

At the time of this work, author's Trinette Chuang and Archana Ramesh worked for the R&D Antibody Development Department of EMD Millipore, CA, United States of America. The authors declare that the research was conducted in the absence of any commercial or financial relationships that could be construed as a potential conflict of interest.

## Supplementary material

Movie 1 **Confocal imaging of a retinal neuron positive for VNUT (red) and fluorescent ATP marker (green) shown over a 30 s interval under basal conditions, when vehicle (PSS) was added at 15 s**. Fluorescence intensity near the membrane was not altered over the recording interval.

Movie 2 **Confocal imaging of a retinal neuron positive for VNUT (red) and fluorescent ATP marker (green) shown over a 25 s interval under depolarizing-conditions, where 55 mM K^+^-PSS was added at 13 s**. Quinacrine fluorescence at the cell membrane reduced significantly within a second after stimulation.

Movie 3 **Another series of confocal imaging of a retinal neuron positive for VNUT (red) and fluorescent ATP marker (green) shown over a 45 s interval under depolarizing-conditions, where 55 mM K^+^-PSS was added at 31 s**. Quinacrine fluorescence at the cell membrane reduced significantly within a second after stimulation.

Movie 4 **Confocal imaging of a retinal neuron positive for VNUT (red) and fluorescent ATP marker (green) shown over a 44 s time interval under depolarizing-conditions in the presence of cadmium Cd^++^ (55 mM K^+^-PSS added at 16 s)**. There was no significant change in fluorescence intensity near the membrane in the presence of cadmium.
